# The case for decolonizing data sources in indigenous evaluation and research

**DOI:** 10.3389/fpubh.2026.1875297

**Published:** 2026-08-12

**Authors:** Allyson Kelley

**Affiliations:** Allyson Kelley and Associates PLLC, Sisters, OR, United States

**Keywords:** American Indian/Alaska native, decolonalization, evaluation, indigenous knowledge and research, participatory action research, research methods (other)

## Abstract

Western public health research and evaluation remain grounded in deficit-based frameworks that often fail to capture the complexity of Indigenous wellbeing. For American Indian and Alaska Native (AIAN) communities, these approaches emphasize measurable disparities while obscuring their root causes. Standard quantitative indicators frequently misalign with Indigenous contexts, overlooking spiritual, relational, and collective dimensions of health. Drawing on the framework of My Story, Your Story, and The Story, this paper highlights how individual, collective, and universal ways of knowing can deepen understanding of health and wellbeing. Decolonizing data and expanding what counts as data is essential to accurately represent Indigenous realities and support meaningful, community-driven change; this work calls for a rebalancing that values Indigenous frameworks alongside conventional methods.

## 1 Introduction: beginning the story

*In fact, almost all of Western science is reductionist in nature and seeks to force natural experience and knowledge into pre-determined categories that ultimately fail to describe or explain anything. The whole process of Western science is that of finding common denominators that can describe large amounts of data in the most general terms, rejecting anything that refuses easy classification as “anomalous,” existing outside the generally accepted labels, and, therefore, not to be given standing or serious attention. This way of gathering information about the world-and ourselves-is, of course, absurd*. —Vine Deloria (1)

There are over 1.9 million American Indians and Alaska Natives (AIANs) and 575 federally recognized Tribes and villages in the United States (2). Each carries its own language, culture, history, and distinct ways of creating, collecting, curating, and sharing knowledge. Despite this diversity, research and health initiatives in Indigenous communities are still largely driven by Western frameworks and methods. The problems with using Western research methods in Indigenous spaces are well-documented (3–5). Western methods often clash with Tribal values, resulting in cultural misalignment, weakened reliability, and limited effectiveness; some examples include reductionist vs. holistic, positivist vs. relational, quantitative vs. qualitative, linear vs. cyclical, and power vs. humility.

### 1.1 Deficit-based data cycles

A key factor in the US public health policy agenda is its reliance on deficits; Indigenous communities must demonstrate problems to receive support. In some cases, the greater the deficit, the greater the funding. This creates cycles of deficit-based research and evaluation that highlight extant needs rather than culturally preferred methods of resilience, strengths, or solutions. It also creates unrealistic federal programming and funding agendas that require AIAN people to address health disparities without addressing the underlying issues that cause them. For example, data from the Centers for Disease Control and Prevention show that AIAN populations experience the lowest life expectancy among US residents (6). These data alone quantify disparity without explaining it. Data do not account for the historical trauma, structural inequities, or the ongoing effects of colonization. Similarly, data from the United States Census report that AIANs experience the highest poverty rate among all racial groups (7), but the poverty data fail to capture the systemic drivers of persistent poverty or convey the underlying structural and social determinants of health (3). Presented this way, data not only obscures root causes but also contributes to data erasure.

The overreliance on Western approaches and evidence in research and evaluation is a challenge within Indigenous communities. Standard indicators of wellbeing, defined, for example, by a number of days from sobriety to relapse, average life expectancy, income level, degree / educational status, offer narrow, incomplete representations of Indigenous health, failing to capture the actual story of Indigenous wellbeing, or in some cases, Indigenous health disparities. The focus on quantitative evidence in large-scale population studies is a mismatch for Indigenous communities and spaces where smaller population sizes, rural communities, and limited resources challenge implementation. These methods also overlook spiritual life, Indigenous knowledge systems, and collective wellbeing. Addressing this requires further decolonizing the positivist paradigm of research (4), which prioritizes hypothesis testing (5), and instead advancing Indigenous-led research in which communities guide design, data collection, interpretation, and reporting (8).

Decolonized data practices honor Tribal sovereignty, engage communities, and center holistic ways of knowing grounded in place, history, culture, language, and worldview (9). The use of community-led, created, curated, and known data in Indigenous health research and evaluation is imperative to fully capture the strengths, resilience, and collective wellbeing of an individual, family, community, or nation. Many Indigenous and non-Indigenous researchers have done this, and more are doing so, but a significant gap in the published Western literature remains regarding how to use decolonized data in public health research in ways that are publishable, equitable, and respected, just as with more standard data sources like surveys, experiments, observations, interviews, and p-values. This perspective article aims to address this gap while providing examples of decolonized data in the Western research milieu.

How do we evaluate and capture dreams? Songs? Visions? Prayers? Wisdom? Or is this part of the unknown, great mystery my Elders tell me about? Are we in a war of wisdom where knowledge is owned, weaponized, monetized, and controlled by someone or something other than ourselves, our stories?

## 2 Seeing what has always been there: decolonized data

Decolonized data can be difficult to define, in part because it is rarely represented in published literature. The Urban Indian Health Institute offers a critical starting point through its decolonizing data toolkit (https://www.uihi.org/resources/decolonize-data-toolkit/), emphasizing that decolonized data must acknowledge the harmful practices of Western researchers and the need for healing, restoration, and reparations. Their definition of decolonized data is: 1) reclaiming the Indigenous value of data collection, analysis, and research, 2) data for Native people by Native people, and 3) recognizing the inherent strengths of Indigenous people. Importantly, decolonized data is strength-based, community-governed, and accountable in all phases of data collection. It elevates cultural knowledge and reflects the immense diversity across Tribal people, communities, and Nations. Decolonized data may take forms that Western frameworks often fail to recognize as “data”: visual art, music, photography, spiritual practice, dreams, and ceremony. They are living evidence of history, reality, identity, language, and culture. Importantly, Indigenous peoples have used data and Indigenous ways of knowing to survive famine, climate change, mass migration, war, and cultural genocide (10). Emerging methods begin to make this visible. For example, approaches such as Photovoice promote the strength and resilience of Indigenous community members and youth (11). Poetic inquiry acknowledges diverse ways of knowing and being while challenging dominant positions of power and perspectives (12). Storytelling, music, songs, talking circles, and other oral traditions are increasingly integrated to elevate Indigenous health research and engage Indigenous people as co-creators of data. (13) Dreams (14, 15), contemplative practices/prayer, and other existentialist and out-of-body (16) data sources may be used within a decolonized setting.

Indigenous Elders and wisdom keepers emphasize balance in all aspects of life, including research and evaluation. Yet current systems remain imbalanced. Western, deficit-based, quantitative models continue to dominate research, evaluation, and funding structures, shaping how health is defined and measured. These models often fail to convey what wellbeing actually looks or feels like in Indigenous contexts. This disconnect is particularly evident in program evaluation. Funding agencies often require the use of Western evidence-based practices or treatments, often derived from non-Indigenous populations and applied to Indigenous contexts. Further, these agencies require Indigenous communities to report success stories, evidence of effectiveness, outcomes, and assessments of success and validity using narrowly defined criteria and data sources. As a result, Indigenous communities must translate their experiences into frameworks that do not reflect their values. Researchers, evaluators, policymakers, funding agencies, students, professors, communities, and health programs are left to determine what kinds of data are valid, reliable, real, and useful when working in AIAN research and evaluation (17). Within these constraints, there is limited space to consider alternative forms of evidence that more accurately represent community realities or health experiences.

### 2.1 Data sources, governance, institutional review boards, and power

Another key factor driving evaluation and research data sources in Indigenous spaces is the role of institutional review boards (IRBs). Despite widespread efforts to establish and implement Tribal IRBs, most research and evaluation efforts continue to be governed by non-Tribal IRBs. Many non-Tribal IRBs call for colonized data approaches and methods, colonized consent forms, data ownership/use agreements, and sufficient power or sample sizes. To obtain funding approval, researchers and evaluators must submit to these regulatory authorities. Some IRBs do not consider the CARE Principles (Collective Benefit, Authority to control, Responsibility, and Ethics) and the importance of self-determination and innovation in data collection and research (18). The lack of acknowledgment of CARE Principles and the emphasis on positivist research methods within the IRB process result in research approaches and data that are not relevant to a community; they lack depth, meaning, and cultural context for use and uptake.

### 2.2 Finding the story: curating decolonized data sources in research and evaluation

Elders and wisdom keepers remind us there are always three or more levels of meaning we must acknowledge in any given story: My Story, Your Story, and The Story (19).

- My Story is the first level of meaning and interpretation; it represents an individual's feelings, personal life, ideas, opinions, and ego.
- Your Story is the second level of meaning that represents the collective experiences of a community, Tribe, Pueblo, village, organization, or place.
- The Story represents the actual truth that connects everything and everyone. Some may say this is an unknowable, unquantifiable, unrealistic quest, or part of the great mystery that we cannot explain with our earthly minds and intelligence. But this is not up for debate or discussion; it simply is.

Western research and evaluation often recognize only fragments of these layers. Decolonized approaches ask us to hold all three simultaneously. Researchers, evaluators, evidence authors, creators, and data curators must keep these three stories in mind when considering data sources and evidence in Indigenous spaces. By acknowledging and integrating these three levels of story into research and evaluation, it is possible to get closer to the truth that connects everything in the universe and everyone. The universal truth of what it means to be human, to heal, to love, and to die. Connecting the universal truth to a deconstructed visual experience is necessary, but presenting these as specific objects or data in Western-published research papers is rare.

### 2.3 Deconstructing and decolonizing data: examples from the field

**Figure 1:**
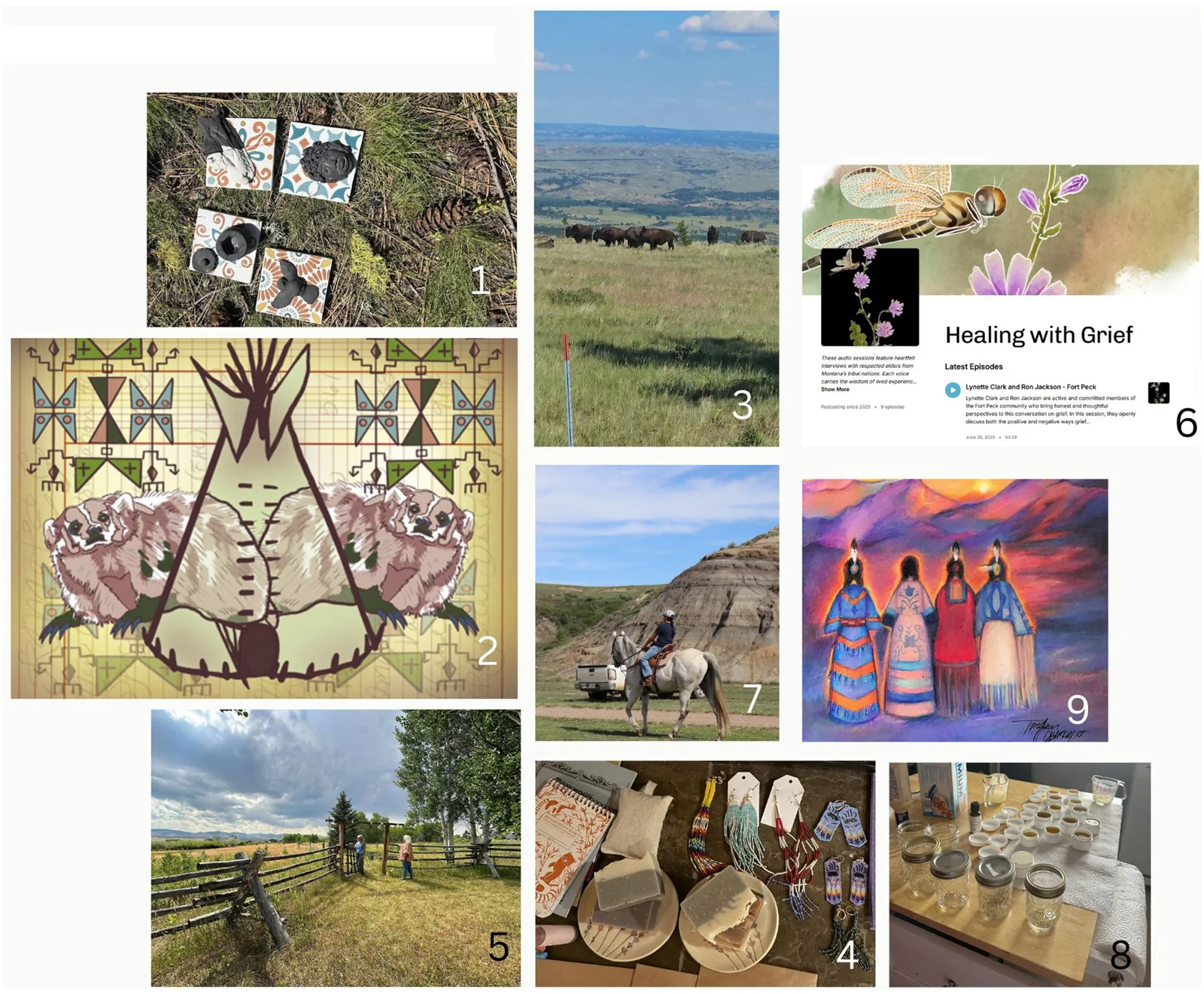
Decolonized data sources.

Decolonized data does not aim to meet colonized standards of objectivity, replicability, validity, or reliability. However, objectivity is not the goal of decolonizing data. In Table 1, I present examples of decolonized data sources using My Story and Your Story as pathways toward The Story or to a universal understanding of a given program evaluation, research question, or phenomenon. I present a deconstructed visual experience, object, or out of body experience that represents a theme or message of wellbeing, informed by my research and evaluation work (http://www.allysonkelleypllc.com) with Indigenous communities and programs. over two decades. Table 1 shows the pathway from My Story and Your Story to The Story using decolonized data sources (Figure 1).

**Table 1:** Decolonized data sources, my story/your story and the story/universal truth.

Decolonized data source	My story/your story	The story
1. Clay figures	^∧^Indigenous good health and wellness in Indian country (GHWIC) team activity, clay representations of heart, mind, body connections, April 2026.	Figures are shared objects, evidence of GHWIC's existence that will live beyond our lifetimes. Federal agencies are funding tribes to address health disparities and value evaluation teams, with impacts that are unknown and unquantifiable.
2. Animal drawings	Representation of teepee teachings and badger in a Tribal Consortium's 5-year evaluation report, April, 2026. Illustration by Tiffany Wolfe, Navajo/Lakota artist.	Animals talk to us, protect us, and watch over us. Their presence in our reality has been documented in oral histories and teachings throughout time.
3. Buffalo pasture	Representation of a Tribe's protection of land/culture at a youth environmental camp, July 2025.	Buffalo signifies the resilience of culture, ecological and spiritual healing, stories, and experiences that live in the minds of youth throughout their lives and in the lives of their grandchildren's grandchildren.
4. Beaded earrings	Representation of Indigenous woman's healing and beadwork, economic sovereignty, March 2026. Earrings by Beth BigFoot, Northern Cheyenne artist.	Symbols, colors, and designs signify the teachings of her ancestors, their prayers, and guidance.
5. Tribal healing ranch	Representation of tribal sovereignty. The Tribe purchased land back from settlers and is now creating healing spaces for families in recovery, July 2024.	The land provides space for healing, ceremony, and programming. It speaks to us, reminding us to take care of it.
6. Tribal podcasts https://sites.google.com/allysonkelleypllc.com/healing-resources/elders-voices?authuser=0	Representation of integrated inter-tribal, Elder/traditional wisdom for healing around grief and loss, August 2025 by Marcus Red Thunder, Chippewa Cree activist and documentary filmmaker.	Voices, stories, laughs, and tears are lodged in the hearts and minds of all who come to find comfort in Elder wisdom. Wisdom lives on as people find healing and help others heal.
7. Tribal horse culture	Representation of community-wide cultural programming using Traditional horse culture teachings, July 2025, by Jaylen Aguilar, Assiniboine and Sioux photographer.	Animals heal us when we come to them. Horses talk to us, carrying our pain, grief, and suffering. A young man guided by the prayers of ancestors, unseen helpers connecting him to opportunities.
8. Traditional medicines	Representation of culture, generosity, the vitality of the land, stories, and Elder knowledge used by staff to share with non-Indigenous researchers and funders, July 2025.	Traditional medicines and ancestral wisdom continue to provide healing, self-determination, and health sovereignty.
9. Tribal art	Representation of Medicine Mountains, female wisdom, original oil by Déaxkaashdishish, Jordan Armajo, Northern Arapaho, used in evaluation report/theme/design July 2023.	Ancestors are watching over the young artist, guiding her, creating opportunities to share her talents and gifts, and instilling cultural pride.
Poem	Reflection of time in community, essence, memory, words of meaning, July 2025.	The significance of living, feeling, and being in community.
Dreams	Revealed messages and meanings through unconscious processing, 2013–2020.	Connecting dream time to consciousness for shared meanings, communication, and guidance.

^∧^The numbers correspond with images in Figure 1. Poem and Dreams are listed in the manuscript narrative.

#### 2.3.1 Poetry

This free-form poem was written by an Indigenous researcher about a visit to the reservation and time spent with a youth organization.

On the Rez

Six years later,

It has not changed

Street chiefs roam

We call them homeless

Dogs prancing down the road

Tongues hanging low, it's 102 degrees

No water in sight

There are signs now

Justice for Brady

MMIW

Find Jane

Signs reminders of unfair and unjust

Crazy Head Springs is the coolest place to be

Clear green water

Turtles lounging on the log

No one for miles

Buffalo roam 15000 acres

They are babies and grandpas

Elder Max tells about the Great Race, buffalo ate people

Magpie saved the people

Kids are not listening

Max's voice, barely audible

The Great Race story

Never heard in this van

#### 2.2.2 Dreams

These dreams are with my mentor, Elder, and teacher, who has gone on to the next camp. He was a veteran, a keeper of knowledge, and a man who made you stop in your tracks when you walked in the room because you knew that he had power, and not the power that comes from status, money, or fame.

August 27, 2013

I was at a big school with Native kids everywhere, and it had a really positive feeling. I was walking around kind of like a stranger, and I ran into Annie, who was working there, and Gordon was kind of in the background; he had some kids around him, and he asked me if I was going to help. It was almost like Annie had not seen him, so I told her, “Your dad was here, and he asked me if I was going to help”. He looked really young. He had a look on his face I remember... it was a look from him, like we knew what we were supposed to be doing and to do it.

August 20, 2014

I was at a church for a graduation ceremony for all the high school and college graduates. I was leaving the church (one that I had never been to), and he was there. I had his cross in my hand (the large gold cross his wife gave me when he passed) and was showing it to him. He was pointing at something in the sky. I don't know what it was, and there were three old bells. We were just starting to talk, though I don't recall what he told me. My partner came in and woke me up (for real). He asked me if he was supposed to wake me up, and I said, “No.” He said, “I saw a light go on and off in the hallway and thought it was you.”

December 2, 2020

I had a dream with you (his wife) and him (my mentor) last night. In this dream, I was telling you that I see him (my mentor) all the time, and you were telling me that you don't. Red was the color of the dream, if that makes sense, like I remember the red… I cannot explain the rest in words.

Date Unknown

I was back at school. He was there; we were doing our work, just as we would in communities or at the office when he was here, still with us in the physical body. I looked out the window in my dream, and there was a huge grizzly bear staring back at me. I was not scared. The grizzly bear held my gaze. I woke up, wondering why I was dreaming about grizzlies. It was him.

Excerpt from my mentor's poetry book (20) shared by his family, after his passing to the next camp. The Big Bear visited him in dreams, too.

The Medicine Dream.

The Big Bear visited me again last night.

His voice could not be ignored the first time, but now it is even more forceful and intimidating.

He expects discipline and commitment from me, and he will not be satisfied with anything less than the Vow that will mark my path to the day that I journey to the other side.

My heart was on overload as the Vision of the Vow merges and defined.

I must be strong and make a sacrifice to protect my family and Tribe.

I feel as the Big Bear, and we are brothers in the knowledge that we must try to protect the old ways and our Peoples (20).

## 3 Reflecting on decolonized data experiences

In this perspective paper, I advocate for the use of decolonized data sources in Indigenous research and evaluation as a pathway to identify The Story; a truth that extends beyond individualistic (My Story) or dualistic (Your Story) methods of knowing.

Clay figures answer process evaluation questions and provide evidence that people gathered on the land and created a tangible object that represents who they are. Animal drawings provide insight into cultural teachings, serving as metaphors for conducting evaluation and presenting results in a decolonized and culturally specific way. Beaded earrings are evidence that programming and partnership can lead to healing and economic stability. Photos of buffalo pastures and a healing ranch show that Tribes are reclaiming their ancestral lands, creating healthy spaces that connect Tribal members to the land, animals, and ancestral teachings. Tribal podcasts by Elders are evidence of integrating Elder wisdom into programs and research.

As with all decolonized data, it is up to the person who generates or collects it to identify My Story, Your Story, and The Story within the data. Importantly, Traditional Knowledge and data are context-specific, connected to land, culture, and Tribal priorities and practices. There are multiple interpretations, meanings, and uses of these data to elevate the health and wellbeing of Tribal communities; in this example, all decolonized data have been used in myriad ways and continue to educate, inform, elevate, and connect people to wellness in published reports, research, or community presentations. Others hang on walls, occupy pages in books, or, in some cases, occupy liminal spaces.

The evidence we draw upon across evaluation, research, community building, retreats, and gatherings is grounded within Indigenous contexts and lived realities. There is an opportunity and a responsibility for Western researchers, evaluators, publishers, editors, policymakers, and funding agencies to become allies in the use of holistic, subjective, place-based evidence and knowledge.

To fully understand and represent wellbeing in Indigenous spaces, we must expand what counts as data. We must elevate multiple ways of knowing and evidence to find The Story. Visual, relational, cultural, and spiritual forms of knowledge are not supplementary; they are essential. Decolonized methods must be taught, shared, and published within Western research spaces. We must decolonize Western research and evaluation to make space for Indigenous knowledge systems, histories, experiences, and evidence, actively reshaping the structures that have historically excluded them.

## 4 Conclusion

These images, dreams, stories, art, and data are offerings to relatives across Turtle Island, inviting a broader understanding of what is real, true, and present. They reflect not one way of knowing, but many. The key lesson I have learned from this collective journey through decolonizing data is that we must continue to seek the truth. We must forget what we know, take a step back, then another, and another, and move intentionally toward new pathways. As evaluators and researchers, we must not overly rely on colonized methods and tools that diagnose, pathologize, subjugate, shame, stigmatize, and generalize human beings. We must consider context through the lens of My Story, Your Story, The Story, and lived experiences, which give them meaning and truth. Use of Western research must not be discarded but balanced with decolonized, Traditional Ways of Knowing, while capturing what is happening within the individual as a whole and, more broadly, within the community.

The dominance of colonized data sources and research methods has created an imbalance in ways of knowing, creating deficit-based models and obscuring strength, resilience, and truth. Our work at AKA, these stories, and this data remind us there is always more to The Story. To achieve meaningful results and create lasting change as we prepare for the next generation, we must pause and listen. We must pray and be intentional about our words, prayers, and the evidence we use to measure what is effective, what constitutes wellness, and what is real.

*I know, and I sense things are changing. My Elder tells me she senses this, too, and fears for our children and grandchildren. “You know, when I was a girl, my grandfather would tell me stories that began… back with the animals would talk to the people… These stories are not being passed down, the animals are no longer talking to the people, and even if they were talking, nobody would be there to listen.”*—Elder Wisdom Keeper, Mentor, 2026

## Data Availability

The original contributions presented in the study are included in the article/supplementary material, further inquiries can be directed to the corresponding author.
